# COX-Independent Mechanisms of Cancer Chemoprevention by Anti-Inflammatory Drugs

**DOI:** 10.3389/fonc.2013.00181

**Published:** 2013-07-11

**Authors:** Evrim Gurpinar, William E. Grizzle, Gary A. Piazza

**Affiliations:** ^1^Department of Pharmacology and Toxicology, The University of Alabama at Birmingham, Birmingham, AL, USA; ^2^Department of Pathology, The University of Alabama at Birmingham, Birmingham, AL, USA; ^3^Drug Discovery Research Center, Mitchell Cancer Institute, University of South Alabama, Mobile, AL, USA

**Keywords:** NSAIDs, cancer, chemoprevention, targets, sulindac, colon

## Abstract

Epidemiological and clinical studies suggest that non-steroidal anti-inflammatory drugs (NSAIDs), including cyclooxygenase (COX)-2 selective inhibitors, reduce the risk of developing cancer. Experimental studies in human cancer cell lines and rodent models of carcinogenesis support these observations by providing strong evidence for the antineoplastic properties of NSAIDs. The involvement of COX-2 in tumorigenesis and its overexpression in various cancer tissues suggest that inhibition of COX-2 is responsible for the chemopreventive efficacy of these agents. However, the precise mechanisms by which NSAIDs exert their antiproliferative effects are still a matter of debate. Numerous other studies have shown that NSAIDs can act through COX-independent mechanisms. This review provides a detailed description of the major COX-independent molecular targets of NSAIDs and discusses how these targets may be involved in their anticancer effects. Toxicities resulting from COX inhibition and the suppression of prostaglandin synthesis preclude the long-term use of NSAIDs for cancer chemoprevention. Furthermore, chemopreventive efficacy is incomplete and treatment often leads to the development of resistance. Identification of alternative NSAID targets and elucidation of the biochemical processes by which they inhibit tumor growth could lead to the development of safer and more efficacious drugs for cancer chemoprevention.

## Introduction

**Figure 1:**
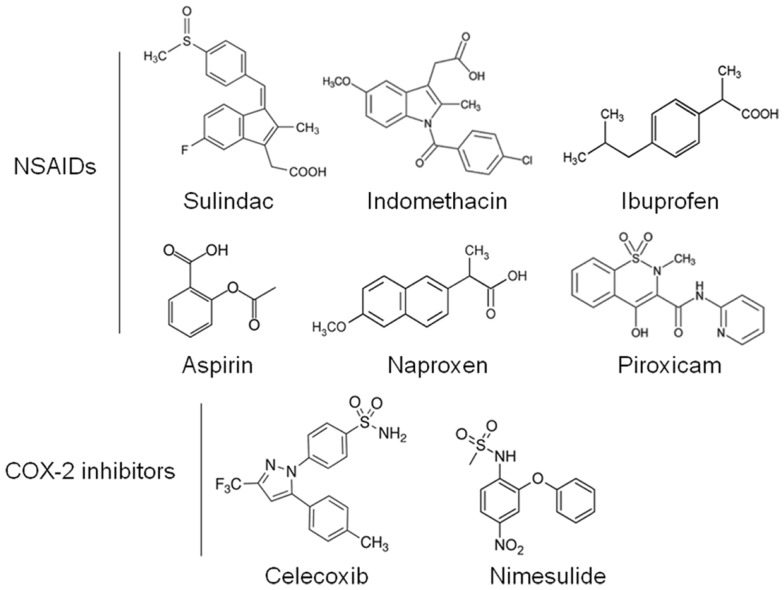
**Chemical structures of common NSAIDs and selective COX-2 inhibitors**.

**Figure 2:**
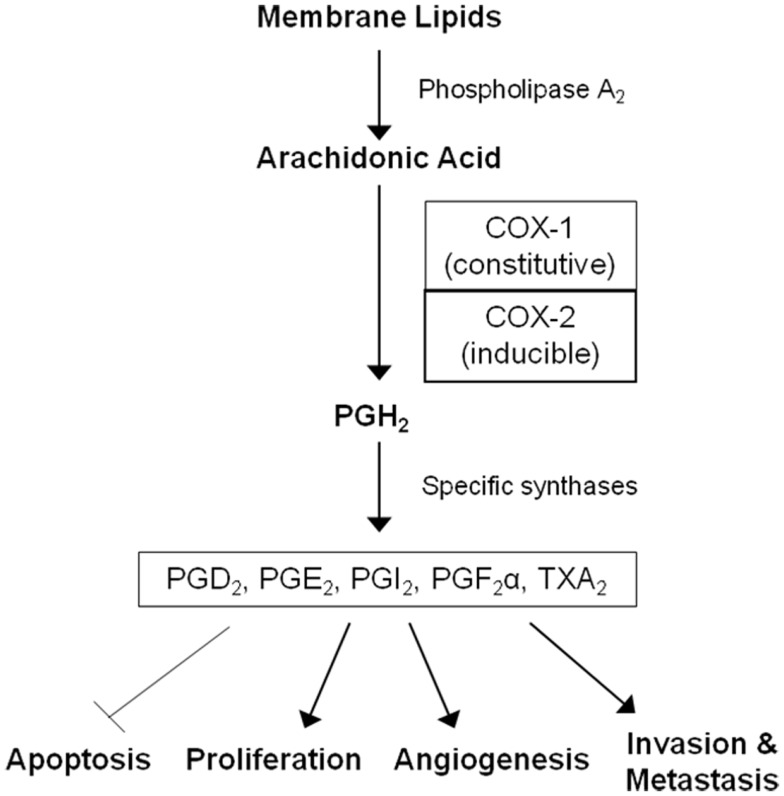
**The arachidonic acid cascade and cancer development**. COX enzymes catalyze the conversion of arachidonic acid into prostaglandin H_2_, the precursor for all prostaglandins (PGs) and thromboxane A_2_ (TXA_2_). PGH_2_ is further converted into PGD_2_, PGE_2_, PGI_2_, PGF_2_α, and TXA_2_ by specific synthases. These molecules mediate inflammation and are also involved in gastrointestinal epithelium homeostasis, platelet activation, and kidney function. Prostaglandins can also promote cell proliferation, angiogenesis, metastasis, and inhibit apoptosis leading to tumor growth.

Non-steroidal anti-inflammatory drugs (NSAIDs) are a diverse class of drugs commonly used for the treatment of inflammatory conditions, analgesia, and fever. Specific indications include arthritis, headaches, menstrual cramps, and mild-to-moderate pain from injuries. Figure 1 shows some of the most commonly used NSAIDs and selective cyclooxygenase (COX)-2 inhibitors. The pharmacological basis for the anti-inflammatory properties of NSAIDs is attributed to inhibition of COX-1 and COX-2 enzymes that catalyze the conversion of arachidonic acid into prostaglandin H_2_, a precursor for the synthesis of prostaglandins, prostacyclins, and thromboxanes. These eicosanoids are known to promote inflammation, pain, and fever (1). In addition, they provide protection for the lining of the stomach and intestines from the damaging effects of acid, promote blood clotting by activating blood platelets, and regulate kidney function. COX-1 is constitutively expressed in many tissues and plays an important role in tissue homeostasis, while COX-2 is induced by inflammatory stimuli and is generally believed to be more involved in pathological processes (2). The prostaglandin synthesis pathway and its relation to tumorigenesis are illustrated in Figure 2.

Epidemiological, clinical, and laboratory studies provide convincing evidence that NSAIDs, including aspirin, non-aspirin NSAIDs, and COX-2 selective inhibitors also have strong antineoplastic properties. The chemopreventive efficacy of NSAIDs against colorectal cancer (CRC) is particularly well-established. For example, numerous population-based studies have shown that regular, long-term users of NSAIDs have a significantly lower risk of colorectal adenomatous polyps and CRC than non-users (3–5). Clinical evidence of activity was first reported in case studies by Waddell and Loughry in 1983 in which administration of the NSAID sulindac (Clinoril^®^) was found to be associated with the reduction of precancerous colorectal adenomas in patients with Garner’s syndrome (6). Later, several clinical trials, including a randomized trial conducted by Giardiello and colleagues reported that sulindac can strongly inhibit the formation of adenomatous polyps and cause regression of existing polyps in patients with familial adenomatous polyposis (FAP) (7–10). Subsequently, the COX-2 selective inhibitor, celecoxib (Celebrex^®^), was reported by Steinbach and colleagues to inhibit adenoma formation in FAP patients (11). This study led to the FDA approval of celecoxib for the treatment of FAP in 1999, but was recently withdrawn for this indication upon request by the manufacturer, Pfizer.

Although there are fewer epidemiological studies on cancers other than CRC, multiple studies demonstrate an association between prolonged use of NSAIDs and lower incidence of or deaths from cancers arising from diverse tissues. These include tumors of breast (12–16), lung (12, 17), prostate (12, 18), bladder (12), ovaries (12, 19), esophagus (12), and stomach (12). Epidemiological and clinical studies are supported by evidence from numerous investigators reporting inhibitory effects of NSAIDs on tumorigenesis in various rodent models, including carcinogen-induced or transgenic models of colorectal, breast, and other types of carcinogenesis (20, 21). Among the earliest reports of the anticancer activity of NSAIDs were publications by Pollard and Luckert who described inhibitory effects of indomethacin on carcinogen-induced intestinal tumors (22, 23). Studies in cell culture models have been numerous as well and suggest that NSAIDs have direct inhibitory effects on tumor cell growth and have been especially useful for defining the underlying mechanism of action.

Unfortunately, suppression of prostaglandin synthesis from COX-1 or COX-2 inhibition is associated with gastrointestinal, renal, and cardiovascular toxicities that limit the dosage and long-term use of NSAIDs for cancer chemoprevention. In addition, currently available NSAIDs and COX-2 inhibitors do not completely protect all individuals from developing cancer. For example, there have been reports of resistant adenomas and breakthrough carcinomas during treatment with sulindac, which highlight the limitations of currently available NSAIDs and COX-2 inhibitors for chemoprevention (24). However, it is not clear if their efficacy limitations are attributable to inherent mechanisms of drug resistance or inability to achieve a sufficiently high dosage, given that the anticancer activity appears to require high dosages administered over extended periods of time, which increase the risk of toxicity.

Evidence for the involvement of COX-2 in colorectal carcinogenesis and its constitutive expression in multiple tumor types has led researchers to postulate that inhibition of COX enzymes, especially COX-2, is responsible for the chemopreventive efficacy of NSAIDs and selective COX-2 inhibitors. Detailed reviews on the role of COXs in tumorigenesis have been previously published (5, 24, 25). However, other studies suggest that COX-independent mechanisms may contribute to, or be fully responsible for their anticancer properties. Identification of alternative targets and additional biochemical processes involved in NSAID activity could lead to the development of safer and more efficacious drugs for cancer chemoprevention. This review examines the biochemical processes associated with the antineoplastic effects of NSAIDs and discusses their COX-independent mechanism of action. Detailed mechanistic analyses that link reported direct non-COX targets with the various cellular effects of this class of drugs are provided. In addition, current and potential future approaches to develop safer and more efficacious NSAID derivatives are outlined.

## COX-Independent Mechanisms and Targets

Numerous studies challenge the theory that COX inhibition is solely responsible for the chemopreventive action of NSAIDs by providing evidence that these effects can be exerted, at least partially, through COX-independent mechanisms. For example, *in vitro* studies have demonstrated that NSAIDs can inhibit proliferation and/or induce apoptosis in multiple tumor cell lines of different origins irrespective of their levels of COX-1 or COX-2 expression (26–29). In addition, the growth-inhibitory activity of NSAIDs cannot be reversed by supplementation with prostaglandins, pointing to a mechanism that is independent of suppressing prostaglandin production (30, 31). Furthermore, there is often a significant discrepancy between the potency of a particular NSAID to inhibit COX-1 and/or COX-2 and its potency to inhibit tumor cell growth *in vitro* and *in vivo*. This is highlighted in Table 1, which shows that various chemical families of NSAIDs display appreciably different potencies to inhibit tumor cell growth, yet there is no correlation between their potency to inhibit growth and potency to inhibit COX-1 or COX-2. For example, the non-selective COX inhibitor indomethacin has much lower antiproliferative activity compared with sulindac sulfide despite having a similar chemical scaffold and an approximately 10-fold lower IC_50_ to inhibit both COX-1 and COX-2 in whole blood COX assays (32). Similarly, while selective COX-2 inhibitors celecoxib and rofecoxib inhibit COX-2 with similar IC_50_ values, celecoxib has much higher antiproliferative activity in both COX-2-positive and COX-2-negative cell lines (33). Other studies confirm these findings through the use of genetic methods by showing that: (1) tumor cells in which expression of COX-2 has been knocked down by antisense cDNA do not display increased apoptosis but remain sensitive to COX-2 inhibitors, (2) the level of knockdown does not affect sensitivity to COX inhibitors, and (3) fibroblasts from COX-1^−/−^, COX-2^−/−^, or COX-1/2^−/−^ knockout mice retain sensitivity to various NSAIDs (34–36).

**Table 1:** **Potency of a panel of NSAIDs to inhibit colon tumor cell growth and cyclooxygenases**.

NSAID	Growth IC_50_^1^	COX-1 IC_50_^2^	COX-2 IC_50_^2^	Serum levels (μM)^3^
Celecoxib	50	>30	2.25	2
Sulindac sulfide	60	1.02	10.4	15
Diclofenac	160	0.14	0.05	6
Indomethacin	180	0.16	0.46	1.4
Piroxicam	900	0.76	8.9	17
Ibuprofen	975	4.75	>30	40
Flurbiprofen	1800	0.44	6.42	53
Aspirin	5000	4.5	13.9	10

*^1^HT-29 human colon tumor cells, 72 h MTS assay (184). ^2^Whole blood COX assays (32). ^3^From therapeutic dosages (185)*.

In general, the concentration of a given NSAID or selective COX-2 inhibitor required to inhibit tumor cell proliferation *in vitro* is much higher than that required to inhibit COX-1 and/or COX-2 activity (37). This is an important consideration since experimental studies in rodent models, as well as clinical studies typically demonstrate chemopreventive efficacy of NSAIDs only at doses higher than those necessary for anti-inflammatory effects. For example, Reddy et al. showed that doses of celecoxib required to decrease incidence and multiplicity of aberrant crypt foci (ACF) in the azoxymethane (AOM)-induced mouse carcinogenesis model reached plasma concentrations of approximately 9 μmol/L, while plasma concentrations of 1.3 μmol/L were sufficient to inhibit adjuvant-induced arthritis (38). Lower doses that reached around 1.8 μmol/L plasma concentrations did not have an effect on ACF development (39). In a randomized, placebo-controlled clinical trial, Steinbach et al. reported that celecoxib caused a significant reduction in colorectal polyp burden in FAP patients at a dose of 800 mg/day but not at the standard anti-inflammatory dose of 200 mg/day bid (11). Additional *in vivo* evidence for COX-independent mechanisms of NSAID chemoprevention is provided by a study in the APC^Min/+^ mouse model of colorectal carcinogenesis. Administration of sulindac dramatically reduced the number of tumors in Min without altering eicosanoid formation (40). Also, increasing the levels of prostaglandin E_2_ and leukotriene B_4_ by dietary arachidonic acid supplementation did not affect tumor number or size. It has to be noted, however, that prostaglandin levels are decreased in the colorectal mucosa of FAP patients with adenoma regression on sulindac (41, 42). These results may explain the modest chemopreventive efficacy of currently available NSAIDs such as sulindac or celecoxib at the anti-inflammatory dosages and highlight the need for more potent and selective inhibitors.

**Figure 3:**
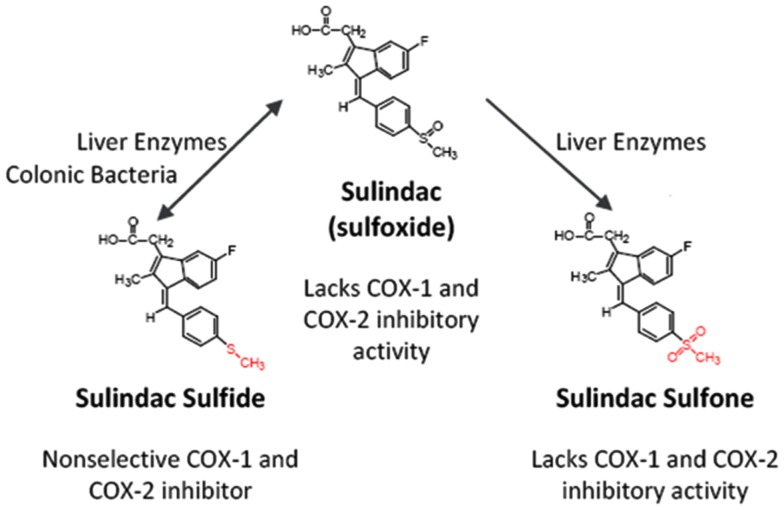
**Metabolism of sulindac**. Prodrug sulindac undergoes reversible reduction into the active sulfide form through the action of liver enzymes and colonic bacteria. Sulindac sulfide is a non-selective COX inhibitor and is responsible for the anti-inflammatory properties of sulindac. The sulfone metabolite is generated by irreversible oxidation of the sulfoxide in the liver, and does not have anti-inflammatory activity. Both sulindac sulfide and sulindac sulfone have antineoplastic activity *in vitro* and *in vivo*.

Perhaps the most compelling evidence that COX-independent mechanisms exist comes from studies showing that NSAID metabolites or derivatives that lack COX-inhibitory activity can retain or have improved antitumor activity. Sulindac sulfone (exisulind) is a prototypical example of a non-COX-inhibitory NSAID derivative with *in vitro* and *in vivo* anticancer activity (43–49). As shown in Figure 3, sulindac is a prodrug that undergoes reversible reduction into the active sulfide form through the action of liver enzymes and colonic bacteria (50). Sulindac sulfide is a non-selective COX inhibitor and is responsible for the anti-inflammatory properties of sulindac. The sulfone metabolite is generated by irreversible oxidation of the sulfoxide in the liver, and does not have anti-inflammatory activity. Numerous studies have shown that sulindac sulfone can inhibit tumor cell growth and induce apoptosis in multiple tumor types despite lacking COX-1 and COX-2 inhibition. Furthermore, sulindac sulfone was shown to effectively inhibit carcinogen-induced tumorigenesis of the colon, mammary glands, lung, and bladder (43–46, 48, 51, 52). In studies involving the AOM model of rat colon tumorigenesis, sulindac sulfone did not reduce prostaglandin levels in the colon mucosa and was able to reach plasma concentrations above those required to inhibit tumor cell growth and induce apoptosis *in vitro* (Table 2). In clinical trials, sulindac sulfone (exisulind, Aptosyn^®^) caused significant regression of polyps in patients with familial (53) or sporadic (54) adenomatous polyposis. Unfortunately, exisulind did not receive FDA approval because of hepatotoxicity, which also limited the dosage.

**Table 2:** **Inhibition of azoxymethane-induced colon tumorigenesis in rats by sulindac sulfone and sulindac (44)**.

Treatment	Dose (ppm)	Tumor burden^#^	PGE_2_ levels (% control)	Serum sulfone (μM)
Control	0	10.5	100	–
Sulfone	500	6.9	94.5	247
Sulfone	1000	3.3*	105	346
Sulfone	2000	1.9*	79	392
Sulindac	400	1.9*	39.5*	121

*p < 0.05, ^#^sum of sizes of adenomas and adenocarcinomas.

Additional evidence to suggest that COX inhibition is not required for the chemopreventive effects of NSAIDs is provided by studies of NSAIDs with a chiral center that exist as a racemic mixture of *S*- and *R*-enantiomers. The *S*-ibuprofen and *S*-flurbiprofen are non-selective COX inhibitors and have antiproliferative activity (55, 56). However, *R*-ibuprofen and *R*-flurbiprofen, which lack COX-1 or COX-2 enzyme activity, also inhibit tumor cell growth *in vitro*, in xenograft mouse models of human tumors, and in transgenic mouse models of tumorigenesis (56–58). Similarly, the *R*-enantiomer of etodolac, devoid of COX inhibition, has been shown to inhibit colorectal carcinogenesis and induce cytotoxicity in multiple myeloma cells (59, 60). It is important to note that about 50% of *R*-ibuprofen is converted to the *S*-enantiomer *in vivo* although the antiproliferative activity does not correlate with COX-2 expression. Synthetic NSAID analogs in which COX-inhibitory activity has been designed-out but retain anticancer activity provide further proof for the existence of COX-independent mechanisms. The sulindac derivatives, SSA and SBA, aspirin derivative, NCX-4016, and celecoxib derivatives, OSU-03012 and dimethyl-celecoxib (DMC), represent some of the non-COX-inhibitory NSAID analogs that display equal or higher antitumor efficacy compared to the parent drug (61–64).

Taken together, these studies provide a strong case that mechanisms independent of COX-1 and/or COX-2 inhibition fully or partially contribute to the chemopreventive activity of traditional NSAIDs and selective COX-2 inhibitors. The anticancer effects of this class of compounds have been proposed to consist of multiple cellular mechanisms, which include induction of apoptosis, inhibition of proliferation and angiogenesis, and more recently, induction of autophagy. A detailed mechanistic analysis of how currently known direct NSAID targets can lead to these biochemical effects is discussed below. These targets are summarized in Table 3.

**Table 3:** **Cyclooxygenase-independent direct cellular targets of NSAIDs and metabolites**.

	Sulindac	Sulindac sulfide	Sulindac sulfone	Celecoxib	Valdecoxib	Aspirin	Salicylate	Indomethacin	*R*-etodolac	Reference
**COX**										
COX-1	–	✓	–	–	–	✓	–	✓	–	
COX-2	–	✓	–	✓	✓	✓	–	✓	–	
**COX-INDEPENDENT TARGETS**										
PDE5		✓	✓	✓						Thompson et al. (71), Tinsley et al. (61), Tinsley et al. (72), Klein et al. (75)
PPARγ								✓		Lehmann et al. (88)
PPARδ		✓						✓		He et al. (92)
RXRα		✓							✓	Kolluri et al. (98), Zhou et al. (100)
IKKβ	✓					✓	✓			Yamamoto et al. (104), Yin et al. (106)
PDK-1				✓						Zhu et al. (111), Arico et al. (112), Kulp et al. (113)
SERCA		✓		✓						Johnson et al. (117), White et al. (123)
CA IX/XII				✓	✓					Weber et al. (128), Di Fiore et al. (129)
Sp1				✓						Wei et al. (157)
AMPK							✓			Hawley et al. (168)

## Induction of Apoptosis

It is now widely accepted that apoptosis is the primary mechanism responsible for the antineoplastic properties of NSAIDs, which was first reported to occur in cancer cells treated with sulindac sulfide by two different groups in 1995 (45, 65). The COX-independent activity of sulindac sulfide was evident by the ability of sulindac sulfone to also induce apoptosis (44, 45). The pharmacological relevance of this effect was demonstrated by studies reporting that treatment with sulindac can stimulate apoptosis in the normal rectal mucosa of FAP patients (66, 67), normal intestinal mucosa of APC^Min/+^ mice (68), and in the colorectal carcinomas of carcinogen-treated rats (69, 70). Interestingly, the non-COX-inhibitory sulindac sulfone was found to induce apoptosis selectively in rectal polyps of FAP patients but not in normal rectal mucosa, which implies an aspect of selectivity not apparent with conventional chemotherapeutic drugs that also act by inducing apoptosis (53). Consistent evidence from *in vitro* studies also demonstrates that traditional NSAIDs and COX-2 selective inhibitors, as well as their non-COX-inhibitory derivatives can induce apoptosis in various cancer cell lines (2, 5, 45). Many mechanisms and targets have been proposed to mediate apoptosis induced by NSAID treatment. While a particular NSAID may have its own, more or less specific, COX-independent target, it is generally recognized that a combination of effects on multiple pathways through direct and indirect targets is responsible for the apoptosis-inducing properties of NSAIDs. Major direct cellular targets that have been shown to mediate apoptosis induction by NSAIDs are discussed below.

### cGMP phosphodiesterases

A direct molecular target for sulindac, celecoxib, and potentially other non-aspirin NSAIDs is the cyclic guanosine monophosphate phosphodiesterases (cGMP PDEs). PDEs are a large family of enzymes that catalyze the hydrolysis of cAMP or cGMP to biologically inactive 5′-nucleoside monophosphates. Previously, it was shown by Piazza and colleagues that sulindac sulfone can inhibit certain cGMP-degrading isozymes, causing an increase in intracellular cyclic GMP levels, thus activating cGMP-dependent protein kinase (PKG), which in turn activates pathways that lead to apoptosis (46, 71). Importantly, a series of sulindac sulfone analogs with improved cGMP PDE inhibitory activity were synthesized and a positive correlation was established between the rank order of potency for PDE inhibition, apoptosis induction, and growth inhibition in colon cancer cell lines (71). More recent studies have shown that sulindac sulfide can also directly bind and inhibit the cGMP-specific PDE5 in recombinant enzyme assays at concentrations lower than its IC_50_ for growth inhibition (61). Although sulindac sulfide was found to have activity against other PDE isozymes, including PDE2, PDE3, and PDE10, sulindac sulfide displayed significantly higher selectivity toward PDE5 inhibition. In addition, PDE5 was found to be overexpressed in various cancer cell lines compared with normal primary epithelial cells. PDE5 appears to be a major cGMP-hydrolyzing enzyme in tumor cells as indicated by the ability of sulindac sulfide to inhibit cGMP-hydrolysis in whole cell lysates and increase intracellular cGMP levels in intact cells. This effect appears to be selective for tumor cells, given that sulindac sulfide more effectively inhibits cGMP PDE activity and induces apoptosis in colon and breast tumor cell lines compared with normal human mammary epithelial cells (HMEC) or colonocytes from normal human colon mucosa (NCM460) (61, 72, 135). Transfecting tumor cell lines with PDE5 siRNA alone was recently found to be sufficient to induce apoptosis and inhibit tumor cell growth (73). Together, these results provide evidence that apoptosis induction by sulindac sulfide is mediated through PDE5 inhibition and the elevation of intracellular cGMP levels. Nonetheless, the contribution of additional PDE isozymes cannot be ruled out, given that conventional PDE5 inhibitors, such as sildenafil, do not display significant potency to inhibit tumor cell growth.

Further testing with a diverse group of other NSAIDs also demonstrated a strong correlation between their ability to inhibit cGMP PDE in lysates from HT-29 colon tumor cells and their growth-inhibitory activity, suggesting that cGMP-specific PDEs could be cellular targets for other NSAIDs as well (72). Indeed, structurally diverse NSAIDs such as celecoxib, indomethacin, and meclofenamic acid were shown to inhibit cGMP PDE activity and increase intracellular cGMP levels in SW480 colon cancer cells (74). Among these, celecoxib was shown to directly inhibit recombinant PDE5 enzyme activity (75), whereas the specific cGMP PDE isozymes that other NSAIDs may bind remains unknown. Interestingly, the COX-2 inhibitor rofecoxib (Vioxx^®^) that was withdrawn from the market because of cardiovascular toxicity and for which anticancer activity has not been well reported, lacks PDE5 inhibitory activity (74). Given that conventional PDE5 inhibitors are being studied for cardioprotective benefits, it is possible that the PDE5 inhibitory activity of celecoxib may reduce its potential for cardiovascular toxicity (76, 77). Consistent with this possibility, sulindac has been previously reported to have cardioprotective benefits in experimental models, despite its COX-2 inhibitory activity (78).

Activation of PKG alone is sufficient to induce apoptosis in colon cancer cells (79) and PKG activation has been shown to occur after treatment with sulindac sulfide, sulfone, and celecoxib (61, 74, 80). One mechanism that activation of PKG can lead to apoptosis in tumor cells is through the suppression of canonical Wnt/β-catenin signaling. We and others have shown that both sulindac sulfide and sulfone can reduce nuclear β-catenin levels, thereby inhibiting Tcf/Lef-mediated transcriptional activity (73). Celecoxib has also been shown to reduce total β-catenin levels and inhibit the DNA-binding ability of the β-catenin/Tcf-Lef complex, although it is unable to decrease nuclear β-catenin levels (81). By contrast, neither rofecoxib nor *R*-flurbiprofen were found to affect β-catenin expression or nuclear localization. The latter compounds are also unable to increase intracellular cGMP levels and activate PKG, pointing to a mechanistic link between cGMP PDE inhibition and inhibition of Wnt signaling that is independent of COX binding. PKG can directly phosphorylate β-catenin in cell-free assays presumably marking it for proteasomal degradation in an APC and GSK3β-independent manner (71). Consistently, sulindac sulfide, sulfone, and celecoxib appear to increase the proteasomal and caspase-dependent degradation of β-catenin (71, 81, 82). In addition, PKG has been shown to attenuate β-catenin mRNA levels by suppressing transcription from the *CTNNB1* gene (83). Indeed, recent results from our lab clearly demonstrate that sulindac sulfide can potently inhibit transcription from the CTNNB1 promoter in colon cancer cell lines resulting in reduced β-catenin mRNA levels. Consistent with PDE5 being a target for these NSAIDs, knockdown of PDE5, by itself, is able to reduce nuclear β-catenin levels and induce apoptosis in breast and colon cancer cell lines (73). These effects were mimicked by use of specific PKG activators such as 8-Br-cGMP and other known PDE5 inhibitors, and are accompanied by reduced levels of anti-apoptotic and pro-proliferative proteins regulated by β-catenin such as survivin and cyclin D1.

**Figure 4:**
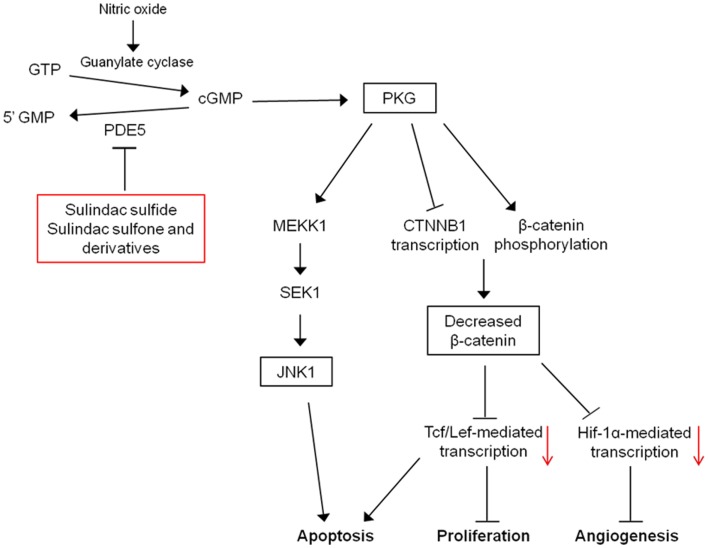
**Mechanistic model for the antineoplastic properties of sulindac**. Inhibition of PDE5 and potentially other PDE isozymes by sulindac metabolites leads to an elevation of intracellular cGMP levels activating protein kinase G. PKG activation can lead to the induction of apoptosis, and inhibition of proliferation and angiogenesis through activation of JNK1 and downregulation of β-catenin-mediated transcription.

An additional mechanism that may mediate the pro-apoptotic effects of PKG is the activation of c-Jun N-terminal kinase 1 (JNK1). PKG can activate JNK1 through phosphorylation of MEKK1 and JNK1 activity and has been shown to be necessary for apoptosis induction by sulindac and sulindac sulfone, *in vitro* and *in vivo* (84). The exact pathway may involve direct phosphorylation and inactivation of anti-apoptotic proteins, Bcl-2 and Bcl-XL by JNK1, and the expression of pro-apoptotic proteins such as Bim or Bad through JNK1-mediated activation of transcription factors (85). In addition, JNK1 has been shown to be necessary for sequestration of β-catenin by FOX04 in the cytoplasm induced by PKG activation (83). These results indicate that inhibition of cGMP PDEs by NSAIDs have the potential to restore APC tumor-suppressor function in colorectal, breast, and potentially other cancer types by attenuating oncogenic Wnt signaling, thereby leading to apoptosis induction. In this respect, PDE5 and possibly other cGMP PDEs represent attractive targets for cancer chemoprevention and/or therapy. A mechanistic model for the induction of apoptosis by cGMP PDE inhibition is provided in Figure 4.

### PPARα, γ, and δ

The peroxisome proliferator-activated receptors are a class of nuclear hormone receptors that regulate proliferation, differentiation, apoptosis, and inflammation by modulating the transcription of a variety of target genes (86). Three isoforms have been identified, PPARα, γ, and δ, all of which bind to specific DNA sequences as heterodimers with the retinoid acid X receptors (RXRs). Loss of PPARγ is implicated in colorectal carcinogenesis, while ligands for PPARγ can suppress breast carcinogenesis in experimental animal models and inhibit anchorage-dependent cell growth in colon cancer cell lines (87). NSAIDs indomethacin (100 μM), ibuprofen (100 μM), fenoprofen (100 μM), and flufenomic acid (100 μM) can activate PPARα and γ in monkey kidney epithelial cells transfected with a PPARγ promoter reporter construct (88). In addition, sulindac sulfide (100 μM) significantly induces PPARγ promoter activity in non-small cell lung cancer (NSCLC) cells, A549 and H2122 leading to increased E-cadherin expression and decreased colony formation in soft agar assays (89). Among these NSAIDs, indomethacin can directly bind purified PPARγ and effectively compete with known PPARγ ligands in cell-free assays. Therefore, it is likely that other NSAIDs are direct ligands for PPARγ as well, although this possibility currently remains untested.

PPARδ, contrary to other PPARs previously described, is a growth promoting protein that is activated by the COX-2 derived prostaglandin, prostacyclin (PGI_2_), and is often overexpressed in colon cancer cells (90, 91). PPARδ expression is negatively regulated by the APC tumor-suppressor pathway through the β-catenin/TCF-lef responsive elements in its promoter (92). As shown by He et al. sulindac sulfide (200 μM) and indomethacin (400 μM) can bind and repress PPARδ transcriptional activity in HCT116 and SW480 human colon cancer cells (92). In addition, overexpression of PPARδ can partially block apoptosis induction after NSAID treatment. Although the authors show that sulindac and indomethacin disrupt the DNA-binding ability of PPARδ/RXR heterodimers leading to inhibition of transcription, it is also possible that a decrease in nuclear β-catenin via cGMP phosphodiesterase inhibition contributes to these effects by directly downregulating PPARδ levels. More recent studies by Liou et al. provide evidence that 14-3-3ɘ, a downstream target of PPARδ, is responsible for its anti-apoptotic effects, and is effectively downregulated by sulindac sulfide, sulfone, and indomethacin treatment in colon cancer cells (93). Overexpression of PPARδ can prevent the reduction in 14-3-3ɘ levels and confer apoptosis resistance, while overexpression of 14-3-3ɘ alone was found to be sufficient to significantly reduce apoptosis levels after NSAID treatment. These findings demonstrate the importance of PPARδ and 14-3-3ɘ as effectors of NSAID-mediated apoptosis and validate their potential as novel targets for cancer prevention and therapy. Furthermore, decreased PPARδ activity cannot be explained by reduced prostacyclin production since these effects are observed in tumor cell lines irrespective of their level of COX-2 expression and also after treatment with the non-COX-inhibitory sulindac sulfone. Novel analogs of sulindac that lack COX inhibition but can activate PPARγ have also been characterized (94).

### Retinoic X receptor-α

Retinoid RXRs are members of the nuclear receptor superfamily involved in controlling many biological processes including carcinogenesis. There are three subtypes of RXR receptors, α, β, and γ, which upon ligand binding heterodimerize with other nuclear receptors such as retinoic acid receptor (RAR), PPARs, liver X receptor (LXR) among others, resulting in DNA-binding and transcriptional activation (95). The relevance of RXRα in cancer is well-established as genetic disruption of RXRα can promote tumorigenesis (96) and RXRα binding to PML/RAR is necessary for the development of acute promyelocytic leukemia (97). The *R*-enantiomer of etodolac, which lacks COX-inhibitory activity, has been shown to bind RXRα and selectively induce apoptosis in tumor cell lines (98). In cancer cells, a truncated RXRα (tRXRα) that results from incomplete proteolytic processing of RXRα also exists, and can act non-genomically through interaction with other proteins to drive tumor cell survival and proliferation (99). More recently, sulindac sulfide was demonstrated to specifically bind tRXRα and inhibit its interaction with the p85α subunit of phosphotidlyinositol-3-kinase (PI3K) (100). This resulted in suppression of downstream Akt signaling and induction of apoptosis across a diverse set of tumor cell lines. A novel derivative of sulindac sulfide devoid of COX-inhibitory activity but with improved potency to bind RXRα (K-80003) was synthesized and shown to have significant antitumor activity *in vitro* and *in vivo*. These effects were significantly attenuated by siRNA knockdown of RXRα indicating that RXRα is a direct target of sulindac, but significantly enhanced by TNFα treatment that was shown to convert Akt signaling to an RXRα-dependent manner in cancer cells. Overall, these findings suggest that RXRα-mediated apoptosis induction contributes to the chemopreventive effects of sulindac, etodolac, and potentially other NSAIDs. The feasibility of targeting RXRα for cancer therapy has already been demonstrated by Targretin, a synthetic RXR ligand, currently in use for the treatment of cutaneous T-cell lymphoma (101).

### IKKβ/NF-κB

Numerous studies provide evidence that NSAIDs may exert their apoptotic effects by directly modulating NF-κB signaling. NF-κB transcription factor, composed of p50 and p65 (Rel A) subunits, is retained in the cytoplasm in its inactive form when complexed with the inhibitory regulatory protein IκB. Phosphorylation of IκB by the PKG IKKβ leads to its ubiquitination and proteasomal degradation leaving free NF-κB to enter nucleus and bind DNA, resulting in transcriptional activation (102, 103). NF-κB mediates its anti-apoptotic effects by activating cellular inhibitors of apoptosis such as TRAF1/2 and c-IAP1/2, or promoters of cell survival such as c-myc. Aspirin (5 mM), sodium salicylate (5 mM), sulindac (1 mM), and its metabolites sulindac sulfide (200 μM) and sulfone (1 mM) can inhibit NF-κB-dependent transcriptional activity in COS cells transfected with an NF-κB-responsive expression vector (104). Furthermore, both *S*- and *R*-enantiomers of flurbiprofen (1 mM), as well as ibuprofen (2 mM) were shown to inhibit NF-κB activity in macrophage and prostate cancer cell lines, respectively (105). Notably, these concentrations are comparable to concentrations required to induce apoptosis in tumor cell lines and the activity of non-COX derivatives such as sulindac sulfone and *R*-flurbiprofen points to a COX-independent effect. Aspirin, salicylate, and sulindac can directly bind and inhibit recombinant IKKβ, the upstream positive regulator of NF-κB (104). In addition, aspirin and salicylate were shown to be potent ATP-competitive inhibitors of this enzyme with IC_50_ values of about 50 μM (106). Therefore, it is likely that inhibition of NF-κB signaling by various NSAIDs also involves direct binding to IKKβ. Consistently, sulindac sulfide and sulfone can inhibit IKKβ enzyme activity in COS cells (104) while celecoxib was found to suppress cigarette smoke condensate-induced NF-κB activation by inhibiting IKKβ phosphorylation in NSCLC cell lines (107).

Dysregulation of NF-κB signaling through mutations in NF-κB itself or in regulatory proteins such as IκB is detected in many tumor types making these proteins attractive targets. However, in some cell types, NF-κB has also been shown to activate apoptosis through a non-canonical pathway. For example, contrary to its effects on NSCLC cells, treatment of cervical cancer cells with celecoxib results in increased NF-κB DNA-binding and apoptosis (108). This discrepancy needs to be addressed when considering targeting this pathway with inhibitors.

### PDK-1/Akt

A direct cellular target for celecoxib is the 3-phosphoinositide-dependent kinase-1 (PDK-1). PDK-1 incorporates growth signaling from upstream PI3K by phosphorylating and activating protein kinase B (PKB/Akt), a critical regulator of cellular proliferation and survival. In many tumors, particularly with PTEN deletions, the PI3K/PDK-1/Akt pathway is constitutively activated promoting tumor growth. Akt exerts some of its anti-apoptotic effects by phosphorylating and inactivating the pro-apoptotic protein BAD, stabilizing β-catenin levels by direct phosphorylation and inactivation of GSK3β or by phosphorylating caspase-9 to prevent its cleavage into active caspase-9, among others (109, 110). Many studies have reported that induction of apoptosis by celecoxib is associated with inhibition of PDK-1 and its downstream target Akt (111–113). In cell-free assays, celecoxib can inhibit recombinant PDK-1 in an ATP-competitive manner (IC_50_ = 48 μM). The importance of these targets for apoptosis induction appears to be dependent on mutational or expressional status of these kinases, as overexpression of PDK-1 but not Akt can confer resistance to celecoxib-induced apoptosis in HT-29 colon cells (112), whereas overexpression of either one of these kinases produces a marginal rescue in viability in PC-3 prostate cancer cells (111). Furthermore, HT-29 cells expressing a kinase-defective PDK-1 remained sensitive to celecoxib. Moreover, inhibition of Akt phosphorylation, the primary target of PDK-1, is not consistently observed across all tumor cell lines even though apoptosis induction is comparable after celecoxib treatment (114, 115).

Although these findings raise questions about the involvement of PDK-1 in celecoxib-induced apoptosis, structurally similar analogs that are more potent inhibitors of PDK-1 but that lack COX-2 inhibition, such as OSU-03012 (111) and DMC (116), have improved apoptosis-inducing and growth-inhibitory activities, implicating PDK-1 in the pro-apoptotic effects of celecoxib. Given the importance of PDK-1/Akt signaling in tumor growth, these compounds represent promising leads that can be exploited for the development of safer and more efficacious derivatives.

### Sarcoplasmic/ER Ca^+2^ ATPase

Sarcoplasmic/ER Ca^+2^ ATPase (SERCA) is a transmembrane endoplasmic reticulum (ER) protein that maintains the Ca^+2^ gradient between the cytosol and the ER. Celecoxib can directly inhibit SERCA activity (IC_50_ = 35 μM) in PC-3 human prostate cancer cells, thereby preventing Ca^+2^ reuptake into the ER and leading to elevated free intracellular Ca^+2^ concentrations (117). It was demonstrated, through the use of microsome and plasma membrane preparations from PC-3 cells, that celecoxib specifically inhibits the ER Ca^+2^ ATPase while exerting no inhibition on the plasma membrane Ca^+2^ ATPase. Intriguingly, this activity was found to be highly specific for celecoxib and was not observed with other NSAIDs including rofecoxib. A number of subsequent studies eventually established that calcium release from the ER is a rapid and potent effect of celecoxib treatment, resulting in the activation of the ER stress response (ESR) (118, 119). The primary purpose of ESR induction is to alleviate the effects of the particular cellular insult and maintain ER homeostasis. However, in conditions of persistent disturbance, such as continued calcium leakage with celecoxib treatment, ESR has been shown to mediate cell death by triggering apoptosis. As a consequence, typical features of ESR can be observed in celecoxib-treated cells. These include global repression of protein translation indicated by phosphorylation and inactivation of eukaryotic translation initiation factor 2α, but strong induction of proteins that mediate ESR such as glucose regulated protein molecular weight 78 (GRP78) and CHOP (CCAAT/enhancer binding protein homologous transcription factor) (120–122). Importantly, ESR-inducing activity is also displayed by the non-COX-inhibitory DMC derivative of celecoxib (119). In this study, ESR activation was observed in mouse xenograft tumors after celecoxib and DMC administration, underscoring the *in vivo* relevance of this pathway in celecoxib-induced apoptosis.

A more recent study has shown that sulindac sulfide can also bind SERCA in a similar fashion to celecoxib, albeit with lower potency (123). This inhibition was associated with elevation of cytosolic Ca^+2^, induction of GRP78, and activation of ER-associated caspase-4 in glioma cell lines. The potency of celecoxib and sulindac sulfide to induce GRP78 correlated with their potency to inhibit glioma cell growth suggesting that ESR activation is involved in their glucotoxicity. Finally, it is worthwhile to further note that sustained elevation of cytosolic Ca^+2^ levels can directly initiate apoptosis irrespective of ESR owing to the critical role of Ca^+2^ in regulating mitochondrial permeability transition pores and Ca^+2^-sensitive endonucleases, proteases, and caspases.

### Carbonic anhydrases

Carbonic anhydrases (CAs) are a large family of zinc metalloenzymes that catalyze the reversible interconversion of carbon dioxide and bicarbonate, thereby regulating acid-base balance in blood and other tissues. At least 12 isoforms have been identified, some of which are cytosolic and others are membrane-bound. Many tumor cells express membrane-bound CAs IX and XII, that are under the transcriptional control of hypoxia-inducible factor-1 (Hif-1) (124). These CAs were shown to promote tumor growth by counteracting acidosis under hypoxic conditions (125, 126). Furthermore, their expression levels correlate with tumor aggressiveness and an extremely poor prognosis (127). Celecoxib, by virtue of its sulfonamide moiety, can bind to the catalytic zinc of CAs and potently inhibit a number of these enzymes (CAs I, II, IV, IX, and XII) in the low nanomolar range (128). The IC_50_ of celecoxib against tumor-associated CAs IX and XII are reported to be 16 and 18 nM respectively (129), values significantly lower than its IC_50_ for COX-2 inhibition (40 nM) (130). These studies also demonstrate that valdecoxib can inhibit these enzymes with comparable potency, whereas rofecoxib, which contains a methyl sulfone group, does not inhibit CA activity.

These findings suggest that celecoxib could induce apoptosis in tumor cells through a mechanism that involves preventing hypoxic adaptation ultimately resulting in reduced intracellular pH and impaired cellular metabolism. Although a number of studies provide strong evidence through use of genetic knockdown methods for the involvement of CAs IX and XII in tumor growth, studies directly implicating these enzymes in celecoxib-induced apoptosis are still lacking. However, several specific CA inhibitors such as acetazolamide, methazolamide, or ethoxzolamide previously have been demonstrated to have significant antitumor efficacy in multiple *in vitro* and *in vivo* models (131), highlighting the need for future studies aimed at determining whether these enzymes represent valid non-COX targets of celecoxib that contribute to its pro-apoptotic properties.

## Inhibition of Proliferation

Unlike their well-defined ability to induce apoptosis, inhibition of proliferation by NSAID treatment is primarily observed *in vitro* or in experimental animal models. Notably, in clinical studies, sulindac caused adenoma regression in FAP patients without affecting proliferation in rectal mucosa (66). Although these findings do not exclude potential antiproliferative effects in adenomas, sulindac sulfone was also shown to lack antiproliferative activity in colorectal polyps of FAP patients (53). Conversely, other clinical studies indicate that celecoxib can decrease proliferation rates in adenomas from FAP patients (132) as well as in the bronchial epithelium of former smokers (133). Animal studies support these observations by providing evidence that various NSAIDs, including sulindac sulfide, can decrease proliferation rates in tumors from carcinogen-treated rats (69) and in APC^Δ716^ mice (134). Indeed, aspirin, sodium salicylate, sulindac sulfide, sulindac sulfone, indomethacin, celecoxib, and piroxicam have all been reported to inhibit cell cycle progression *in vitro* by inducing a G_0_/G_1_ cell cycle arrest (2).

**Figure 5:**
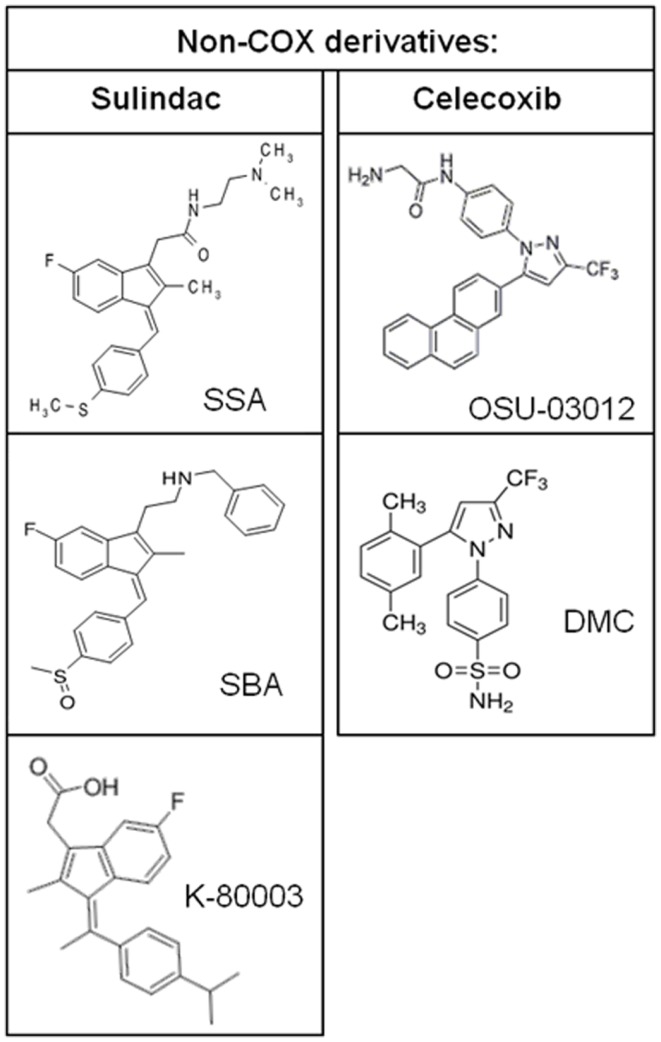
**Chemical structures of non-COX-inhibitory derivatives of sulindac and celecoxib**.

Transitions between different phases of the cell cycle are controlled by various cyclins, cyclin-dependent kinases (CDKs), and CDK inhibitors. Studies from our lab have shown that direct inhibition of PDE5 by sulindac sulfide can downregulate the expression level of G_1_/S transition-specific cyclin D1 in breast cancer cell lines (61). More recent results extend these findings into colorectal adenoma and carcinoma cell lines suggesting that PDE5 inhibition can directly attenuate proliferation in tumor cell lines (135). Since cyclin D1 is a target gene for the β-catenin/TCF-Lef complex, the proposed mechanism of cyclin D1 downregulation was shown to involve elevation of cGMP, activation of PKG, and subsequent attenuation of nuclear β-catenin levels via proteasomal degradation and transcriptional inhibition (Figure 5). Previously, sulindac sulfone and its analogs with improved PDE5 inhibition were also shown to downregulate cyclin D1 levels (136), suggesting that the ability to block cell cycle progression by sulindac metabolites is COX-independent and at least partially mediated through PDE5 inhibition. Another target gene of the APC/β-catenin pathway important for cellular proliferation is the c-myc oncogene, which upregulates cyclins but downregulates p21. Therefore, it is logical to suggest that inhibition of c-myc expression by targeting PDE5 is an additional mechanism by which sulindac can reduce tumor cell proliferation. Importantly, upregulation of the CDK inhibitor p21^waf1^, but not p27^kip1^, was established to be required for the *in vivo* efficacy of sulindac by the finding that tumors in APC^1638N^ mice with p21 inactivation (APC^+/−^, p21^+/−or −/−^ mice) were unresponsive to sulindac treatment (137–139). Later studies demonstrated that the induction of p21 and inhibition of proliferation after sulindac treatment, *in vitro* and *in vivo*, was dependent on the expression of JNK1 (84). These findings provide a potential link between the activation of PKG by sulindac metabolites and the induction of cell cycle arrest via consequent upregulation of JNK1 and p21. Other studies have shown that p21^waf1^ upregulation by sulindac treatment is partially mediated through the COOH-terminal Src kinase (Csk), pointing to the involvement of Csk/Src pathway in the antiproliferative effects of sulindac (140).

The G_1_-phase cell cycle arrest induced by celecoxib treatment in multiple tumor cell lines is accompanied by decreased expression of cyclins A, B, and D; and increased expression of cell cycle inhibitors p21^waf1^ and p27^kip1^ (141–143). One of the targets that can mediate these effects is proposed to be protein kinase B (PKB/Akt) or its upstream kinase PDK-1. As mentioned previously, celecoxib and its COX-sparing analog OSU-03012, can directly inhibit PDK-1 in an ATP-competitive manner thereby blocking signaling through the downstream Akt pathway (111, 113). Akt acts a positive regulator of cell cycle progression by phosphorylating and inactivating CDK inhibitors p21^waf1^ and p27^kip1^. This results in the activation of various cyclin-CDK complexes leading to DNA replication and proliferation (144–146). Therefore, inhibition of PDK-1/Akt signaling represents one mechanism by which celecoxib induces a cell cycle block. Although sulindac sulfide has been shown to inhibit Akt signaling by directly binding RXR, it remains unclear whether its effects on p21 levels and cell cycle progression are mediated through this pathway. However, p21 induction by sulindac treatment is likely not mediated by p53, the well-known positive regulator of p21, since p53 induction is not a determinant of sensitivity to sulindac metabolites in colon cancer cell lines (51).

It is tempting to suggest that inhibition of SERCA by celecoxib and sulindac sulfide is an important mechanism responsible for the antiproliferative properties of these compounds. As a result of ESR-mediated downregulation of general translation, short-lived proteins, such as various cyclins, quickly disappear and cannot be replenished. This leads to the loss of CDK activity for which they are essential subunits. Consequently, CDK target proteins, most notably the retinoblastoma (Rb) tumor-suppressor protein, cannot be phosphorylated allowing them to remain active and prevent cells from progressing toward the S phase. Although a functional link is difficult to establish due to the presence of multiple upstream events, the effects of celecoxib and sulindac sulfide on SERCA provide a plausible explanation for their effects on the cell cycle.

Lastly, direct inhibition of IKKβ by aspirin, salicylate, and sulindac may be another mechanism that could account for the antiproliferative effects of these NSAIDs via downregulation of NF-κB-mediated proliferative genes such as interleukin-2 (IL-2), granulocyte-macrophage colony-stimulating factor (GM-CSF), c-myc, cyclin D1, and COX-2, among others.

## Inhibition of Angiogenesis, Tumor Cell Invasion, and Metastasis

It is now well-established through a large body of evidence that COX-2 derived eicosanoids (prostaglandins, thromboxanes, and leukotrienes) contribute to tumor development through their role in angiogenesis. COX-2 overexpression in tumor cells, surrounding stroma, and/or interstitial inflammatory cells (predominantly macrophages) promotes tumor vascularization by inducing the expression of vascular endothelial growth factor (VEGF) and increasing endothelial cell proliferation and migration (25, 147, 148). Consequently, traditional NSAIDs as well as selective COX-2 inhibitors have been shown to inhibit tumor growth through antiangiogenic mechanisms in experimental models (148). However, several studies provide evidence that COX-independent mechanisms may contribute to the antiangiogenic effects of NSAIDs. For example, sulindac sulfone was found to inhibit angiogenesis in intradermal lung tumor xenografts in mice (149), in the *ex vivo* chick embryo chorioallontoic membrane (CAM) assay (150, 151) and in an *in vivo* mouse corneal neovascularization assay (152). It is important to note that the CAM and corneal neovascularization assays represent non-inflammatory angiogenesis models and hence, the efficacy of sulindac metabolites points to an underlying mechanism unrelated to COX-2 inhibition.

As for potential alternative targets, it is plausible that cGMP phosphodiesterase inhibition by these compounds and subsequent activation of PKG is responsible for their antiangiogenic properties, given the ability of sulindac sulfone to inhibit angiogenesis. In support of this possibility, studies by Browning et al. have demonstrated that PKG overexpression in SW620 colon cancer cells significantly inhibits angiogenesis and reduces VEGF levels after subcutaneous implantation in mice (153). In addition, ectopic PKG expression was shown to block hypoxic adaptation in SW620 xenografts by inhibiting the transcriptional activity of Hif-1α, a critical driver of VEGF expression and angiogenesis (154). Although the mechanism of Hif-1α inhibition by PKG is not fully elucidated, an earlier study by Kaidi et al. provides evidence that β-catenin can enhance Hif-1-mediated transcription through a direct binding interaction (155). In addition to these findings, SW620 cells with PKG overexpression show reduced levels of β-catenin compared with parental cells. Therefore, cGMP PDEs represent potential targets that can mediate the antiangiogenic properties of NSAIDs through attenuation of β-catenin levels (Figure 5). It needs to be considered, however, that the PDE5 inhibitors, sildenafil, and tadalafil, can promote angiogenesis in various models of tissue damage and wound healing (156). Nonetheless, functional consequences of cGMP PDE inhibition on angiogenesis are yet to be tested in neoplastic models.

Studies using human PANC-1 pancreatic adenocarcinoma cell lines indicate that the transcription factor Sp1 may have a direct role in the inhibition of angiogenesis by celecoxib. Constitutive activation of Sp1 is a crucial determinant of VEGF overexpression in pancreatic cancers. Wei et al. demonstrate that celecoxib potently downregulates Sp1 protein levels and inhibits its transactivating activity in PANC-1 cells (157). By conducting deletion and mutational analyses of the VEGF promoter, Sp1 binding sites were shown to be required for celecoxib-mediated attenuation of VEGF expression. In cell-free assays, celecoxib can inhibit the DNA-binding ability of Sp1, suggesting a direct interaction may be responsible for the effects of celecoxib on Sp1 activity. In addition, celecoxib, and other COX-2 inhibitors such as nimesulide and NS-398 can induce proteasomal degradation of Sp1 and decrease Sp1 phosphorylation, in multiple colon cancer cell lines, which is required for its transcriptional activity (158). Although the precise mechanisms responsible for these effects remain unknown, comparable responses were observed in COX-2-expressing and -non-expressing cell lines, suggesting that mechanisms unrelated to COX may be involved.

Evidence implicating a direct NSAID target in the Hif-1α/VEGF-mediated angiogenic response in human tumors is thin. As such, it needs to be considered that the antiangiogenic properties of NSAIDs can be secondary to their effects on endothelial cell survival, proliferation, and/or migration. For example, inhibition of angiogenesis by sulindac sulfide and sulfone in the CAM model was paralleled by induction of apoptosis (150). Similarly, celecoxib and the COX-2-inactive analog, dimethyl-celecoxib (DMC), were shown to inhibit the proliferation of human umbilical vein endothelial cells (HUVEC) and display efficacy in the CAM model at concentrations lower than those required to induce apoptosis (159). These effects were associated with the induction of a G1-phase cell cycle arrest and inhibition of PDK-1/Akt signaling. More recent studies implicate SERCA inhibition as a potential contributor by showing that DMC causes ESR-mediated cell death in tumor-associated brain endothelial cells (TuBECs) (160). As support for this possibility, Neiderberger et al. reported that celecoxib-induced cell death in HUVEC cells is accompanied by elevation of intracellular Ca^+2^ and activation of caspases (161). In addition, rofecoxib was unable to induce apoptosis in HUVECs indicating the involvement of a celecoxib-specific COX-independent target.

Matrix metalloproteases (MMPs) represent another class of major contributors to angiogenesis and invasion that are inhibited by NSAID treatment. MMPs 2 and 9 are the principal enzymes involved in degrading type IV collagen of the basement membrane allowing endothelial cells to reach incipient tumors or cancer cells to invade adjacent tissue leading to metastases (162). In U87 MG human glioblastoma cell lines, celecoxib (40 μM), sulindac (300 μM), sulindac sulfide (150 μM), and sulindac sulfone (400 μM) have been shown to inhibit invasion through downregulation of MMPs 2 and 9 (163). This anti-invasive activity could be abrogated by overexpression of phosphorylated Akt using a Myr-Akt vector or potentiated through the use of a dominant-negative Akt (DN-Akt) vector. Furthermore, downregulation of MMP levels was found to be due to the inhibition of Akt-mediated transcription in glioblastoma cell lines. Although these findings do not provide a definitive target, it can be postulated that celecoxib, through its direct inhibition of PDK-1, and sulindac, via its interaction with RXRα, are able to downregulate Akt-dependent tumor cell invasion. Intriguingly, NSAIDs that have not been previously reported to inhibit Akt signaling such as aspirin, ketoprofen, and naproxen were found to lack anti-invasive properties in these cell lines.

Finally, a recent report by Li et al. provides evidence that sulindac sulfide (50 μM) can inhibit tumor cell invasion by suppressing Nf-κB-mediated transcription of microRNAs in human colon and breast cancer cell lines (164). The majority of microRNAs modulated by sulindac sulfide treatment were found to contain Nf-κB binding sites in their promoter regions. In addition, Nf-κB could be isolated from the promoters of several microRNAs, such as miR-10b, downregulated by sulindac sulfide treatment and previously shown to have a role in tumor cell invasion. Furthermore, sulindac sulfide was able to significantly block the invasion stimulated by the overexpression of these microRNAs. Notably, anti-invasive activity was observed at concentrations lower than those required to significantly inhibit the growth of these cell lines, which suggests potential benefits for preventing metastasis in patients at risk of disease progression. These effects were associated with inhibition of IKKβ and IκB phosphorylation, and attenuation of nuclear Nf-κB levels. Although it is unclear whether these effects are mediated by direct IKKβ binding, this study establishes a mechanistic link between inhibition of Nf-κB signaling by sulindac and its anti-invasive effects via microRNA modulation. Future studies are warranted to test whether aspirin and/or salicylate can replicate these effects since IKKβ is a direct target of these compounds.

## Induction of Autophagy

Autophagy is a catabolic process whereby cells degrade cytoplasmic components in lysosomes. This process is crucial for maintaining cellular homeostasis by removing damaged proteins and organelles, eliminating invading pathogens, and recycling cellular building blocks thereby providing substrates for energy generation. In the past decade, multiple studies have provided genetic and functional links between impaired autophagy and cancer suggesting that autophagy can serve as a tumor-suppressor mechanism. Conversely, consistent with its role in damage mitigation, autophagy has been demonstrated to promote growth of established tumors under conditions of hypoxia, and in response to chemo- or radio-therapy. In certain cases, autophagy has also been shown to mediate cell death either directly through excessive degradation of the cytoplasm or selective digestion of vital organelles such as mitochondria, or indirectly via triggering apoptosis. Excellent reviews on the role of autophagy in cancer have been previously published (165–167).

Regarding NSAID chemoprevention, a growing number of studies report that various NSAIDs and/or NSAID analogs can induce autophagy in tumor cell lines. An interesting connection between aspirin and autophagy was established in a recent report by Din et al. who showed that aspirin (5 mM) can inhibit Akt/mTOR signaling, activate AMP-activated kinase (AMPK), and induce autophagy in multiple colon tumor cell lines, in mice, and in the rectal mucosa of patients on a daily aspirin regimen. Activation of AMPK by aspirin was found to be comparable to that produced by known activators of this kinase, phenformin and metformin. An independent study by Hawley and colleagues concomitantly showed that AMPK is a direct molecular target of salicylate, the active metabolite of aspirin using purified kinase assays (168). These findings are important in the context of numerous epidemiologic, clinical, and experimental studies which indicate that use of metformin, a commonly prescribed antidiabetic drug, is associated with reduced cancer incidence and mortality (169, 170). Furthermore, its antineoplastic activity has been attributed to activation of AMPK and subsequent inhibition of mTOR activity, which among others, results in induction of autophagy (171, 172). Therefore, it is reasonable to suggest that activation of AMPK and subsequent autophagy induction by aspirin may contribute to its chemopreventive effects.

Another report demonstrates that sulindac sulfide can induce autophagy, followed by cell death, in gastric cancer cells at physiologically relevant concentrations (10 μM) (173). Tumor cell death was found to be dependent on downregulation of survivin, which could be abrogated by siRNA knockdown of the essential autophagy protein LC3. These results suggest that survivin may be selectively degraded by autophagic vacuoles after sulindac sulfide treatment. As such, sulindac sulfide-induced autophagy may function as an intermediate process that enables later apoptotic events by preventing the accumulation of an anti-apoptotic protein. As evidence for a COX-independent mechanism, we have recently reported that a novel non-COX-inhibitory sulindac derivative, sulindac sulfide amide (SSA), inhibits the growth of human lung adenocarcinoma cell lines primarily through the induction of autophagy (174). SSA also induced apoptosis, albeit at concentrations appreciably higher than its IC_50_ value for growth inhibition. Nonetheless, treatment with pan-caspase inhibitor z-VAD-FMK could not prevent cell death, whereas suppressing autophagy by Atg7 siRNA could significantly increase cell viability after SSA treatment. Furthermore, the induction of autophagy and loss of cell viability was found to be mediated by Akt/mTOR inhibition and could be partially blocked by the overexpression of activated Akt via a Myr-Akt plasmid. On the other hand, sulindac sulfide only induced autophagy at concentrations higher than those required to inhibit tumor cell growth and apoptosis appeared to be the primary mechanism of cell death. These findings suggest that SSA and sulindac sulfide may share similar targets that ultimately lead to autophagy induction, but that SSA represents a much more potent inducer of autophagy than the COX-inhibitory sulfide metabolite of sulindac. The *in vivo* relevance of the induction of cell death through autophagy by SSA is still untested, but remains an interesting possibility to explain its tumor-selective effects and strong antitumor efficacy as reported in the human HT-29 xenograft mouse model (175). Together, these findings provide proof-of-concept evidence that COX-independent targets of sulindac and possibly other NSAIDs are relevant for the induction of autophagy. As such, celecoxib and its non-COX-inhibitory analog, OSU-03012, are the only NSAIDs known to directly target a negative regulator of autophagy signaling, PDK-1. Nonetheless, the relevance of PDK-1/Akt inhibition in autophagy induction by these compounds is unclear since OSU-03012 has previously been shown to induce autophagy and cell death without affecting Akt phosphorylation (176).

Altogether, the amount of data providing a mechanistic understanding of the precise role of autophagy after NSAID treatment is sparse. Studies described above suggest that induction of autophagy may contribute to the chemopreventive properties of NSAIDs. However, several other studies demonstrate that upregulation of autophagy by NSAID treatment can delay or inhibit apoptosis induction in tumor cell lines (177, 178). The most accurate approach for assessing the role of autophagy in NSAID chemoprevention would be a tumorigenesis model, whereas experiments in cell culture or on established xenograft tumors might reflect conditions in which autophagy will serve to counteract NSAID cytotoxicity. Functional evidence linking autophagy induction and inhibition of tumor formation by NSAIDs in genetic or carcinogen-induced animal models of tumorigenesis is lacking. In order to conclusively answer this question, new mouse strains that harbor mutations in both a tumor-suppressor gene and an autophagy gene such as APC^Min/+^, ATG5^−/−^ or APC^Min/+^, Beclin^−/+^ mice need to be generated.

## Conclusion and Future Directions

Studies of the pathways by which NSAIDs inhibit carcinogenesis have not provided conclusive evidence of the molecular targets that are clinically relevant to their chemopreventive activity. Evidence described above provides a strong case that inhibition of COX enzymes cannot explain the complex antineoplastic activity of NSAIDs. Identification of COX-independent targets and mechanisms most important for the antineoplastic properties of these drugs can be used to develop more efficacious chemopreventive drugs without the gastrointestinal, renal, and cardiovascular side effects associated with NSAIDs and COX-2 inhibitors. Currently, sulindac derivatives have been developed that inhibit PDE5 and have antitumor activity without inhibiting COX-1 or COX-2 (179). These experimental agents demonstrate the feasibility of developing safer and more efficacious drugs for chemoprevention by targeting PDE5. Furthermore, the sulindac derivative K-80003 that has been designed to selectively target RXRα and celecoxib derivatives developed to inhibit PDK-1 without COX-inhibition represent other examples of separating COX-inhibitory activity and antitumor efficacy. Previously published non-COX-inhibitory derivatives of sulindac and celecoxib are shown in Figure 5.

It needs to be stated that most of the studies described in this review were performed using human cancer cell lines and their *in vivo* significance is yet to be determined. The concentrations of NSAIDs used in cell culture experiments often significantly exceed maximum plasma levels that can be achieved in patients in clinical studies. However, despite relatively low blood levels, NSAIDs are able to achieve efficacy *in vivo* while these concentrations are not sufficient to inhibit tumor cell growth *in vitro*. This apparent discrepancy suggests that the *in vivo* response to NSAID therapy may reflect accumulative effects in tissues as a result of chronic administration to patients. Indeed, there is evidence that colonic epithelial cells are exposed to much higher concentrations of sulindac sulfide than those detected in plasma, owing to its unique metabolism by colonic bacteria (180–182). Other studies show that antiproliferative activity can be achieved *in vitro* at clinically relevant concentrations by increasing the duration of treatment. For example, Patel et al. have reported that low-dose (2.5–10 μM) celecoxib treatment can inhibit the growth of COX-2-negative PC-3 and LNCAP human prostate cancer cell lines when treatment period is extended to 96 h (183). Altogether, these findings highlight the need for more detailed studies on tissue pharmacokinetics and tumor uptake of various NSAIDs. In this respect, it is possible that dose-limiting toxicities from COX-inhibition prevent complete response during NSAID therapy and lead to a pharmacodynamic mechanism of resistance, which can be overcome by the optimization of non-COX targets.

Given the complexity and the multitude of pathways that mediate the biochemical effects of NSAIDs, it is highly challenging to identify relevant targets suitable for cancer chemoprevention. Treatment with a single drug can lead to pleiotropic effects by targeting multiple cellular molecules and pathways, while specific targeting of a single molecule can also induce a wide variety of changes in cellular functions. Therefore, it will be useful to consider targeting pathways that show a higher apoptosis-inducing or antiproliferative activity with the least potential for off-target effects. In addition to tissue culture studies, it is crucial to validate the *in vivo* roles of candidate targets of chemoprevention in experimental animal studies for an accurate assessment of efficacy and toxicity. Further elucidation of COX-independent NSAID targets has the potential to contribute to future chemopreventive strategies by enabling identification of novel agents and/or driving rational modification of existing chemopreventive drugs.

## Conflict of Interest Statement

The authors declare that the research was conducted in the absence of any commercial or financial relationships that could be construed as a potential conflict of interest.
